# Nasal Discharge Eosinophils in Childhood Asthma Patients as a Predictive Factor for Persistent Asthma

**DOI:** 10.1155/2018/2563978

**Published:** 2018-12-18

**Authors:** Norihide Murayama, Kikuno Murayama

**Affiliations:** Murayama Pediatrics, Osaka, Japan

## Abstract

**Objective:**

Eosinophilic inflammation is thought to play a role in childhood asthma. Thus, examination of nasal eosinophils, instead of sputum, may be useful for the diagnosis of not only eosinophil-positive rhinitis but also persistent asthma. Nasal eosinophil examination is a routine for the diagnosis of nasal eosinophil-positive rhinitis in patients with rhinorrhea symptoms. This retrospective study investigated whether testing for nasal discharge eosinophils is useful for diagnosing childhood allergic asthma and whether nasal discharge eosinophils are predictive of persistent asthma.

**Methods:**

Infants and young children (*n* = 180) with at least 3 recurrent episodes of respiratory symptoms and bronchodilator inhalation improvements at intervals of ≥1 week were divided into the asthma group and the nonasthma group, and the presence or absence of nasal discharge eosinophils was examined by age. Correlations between nasal discharge eosinophils and other predictive factors for persistent asthma were also examined.

**Results:**

The evaluation of nasal discharge eosinophils in the asthma group showed a significantly higher positive rate in older age groups than in the 0–1-year-old age group (*p* < 0.05–0.001). However, none of the asthma patient groups had any significant changes between the 0–1-year-old and older groups. This pattern was similar for other risk factors, showing correlations between nasal discharge eosinophils and other predictive factors.

**Conclusions:**

Testing for nasal discharge eosinophils with asthma symptoms increases with age. Nasal discharge eosinophils with asthma symptoms may be a predictive factor for persistent asthma. This trial is registered with UMIN Clinical Trials (registration number UMIN000030776).

## 1. Introduction

Airway eosinophilic inflammation has long been known to be associated with asthma [1]. In adult asthma patients, the presence of eosinophils in induced sputum samples and transbronchial biopsies, together with airway hypersensitivity, an obstructive pattern on respiratory function tests, and serum antibodies to allergens, is used to diagnose asthma [2, 3]. However, in childhood asthma patients, induced sputum samples, transbronchial biopsies, and respiratory function tests are difficult to perform. The total IgE Radio Immuno-Sorbent Test (RIST) normal range increases by age to 20 (<1 years), 30 (1–3 years), 110 (4–6 years), and 170 IU/mL (>7 years) (FALCO Biosystems Ltd., Kyoto, Japan). Radio-Allergo-Sorbent Test (RAST) data, as part of the total IgE quantification, has the possibility of increasing at older age, even if patients were negative at a younger age. Although on becoming persistent (allergic) asthmatics, positive (>0.35 UA/mL) RAST rates are also lower in younger children. In addition, the differential diagnosis from nonallergic causes, such as respiratory infections [1] and Gastro-Esophageal Reflux Disease (GERD) [4], is challenging since they can also induce coughing, wheezing, and dyspnea.

This study evaluated nasal discharge eosinophils in infants and young children with both nasal symptoms (without fever) and recurrent respiratory symptoms to evaluate its utility as a potential predictive factor for persistent asthma. Other reported predictive factors for asthma were also evaluated, including peripheral blood RAST positivity [5], allergic family history (relatives within the second degree), and other coexisting or past allergic diseases such as eczema and allergic conjunctivitis.

## 2. Materials and Methods

Nasal eosinophil examinations are routine for patients with continuous nasal discharge symptoms (more than one week), for diagnosing nasal eosinophil-positive rhinitis. We extracted this data from such examinations according to patient age. This retrospective study included patients treated at Murayama Pediatrics at least 3 recurrent episodes of respiratory symptoms such as coughing, wheezing, and dyspnea and an improvement using bronchodilator inhalation at intervals of 1 week more than three times. According to the Japanese guidelines for child asthma [6], which attaches importance for the early intervention of infant or child asthma, these patients were divided into the asthma group (A group) and the nonasthma group (NA group). A total of 180 patients showed nasal symptoms, such as nasal discharge for 1 week, without fever, resulting in 85 patients in the A group and 95 patients in the NA group.

Nasal discharge samples were obtained with a cotton swab from the nasal cavity, smeared onto a glass slide, and stained with Wright Giemsa to determine the presence of nasal discharge eosinophils. The presence of at least 1 eosinophil in any of 10 fields at 400x magnification using a light microscope was defined as positive for nasal discharge eosinophils. Testing was performed by a laboratory technician at the Japan Clinical Laboratories Inc. (Sakai, Osaka, Japan).

Statistical analysis using the chi-square test was performed with JSTAT for Windows version 17.1. The study was conducted at Murayama Pediatrics from August to December 2008. This study was approved by the staff of Maruyama Pediatrics. Informed consent was received orally or in written form from all patients and/or their guardians.

## 3. Results

Table 1 shows the distribution of patients by age in the asthma (A) and nonasthma (NA) groups.  Table 2 shows the distribution of nasal discharge eosinophil counts in both study groups. The nasal discharge eosinophil counts in the entire cohort were negative (−) in 60% of patients, ± in 12.9%, 1+ in 20.0%, 2+ in 2.3%, and 3+ in 4.7%. Most of the positive results were graded as either ± or +.

**Figure 1:**
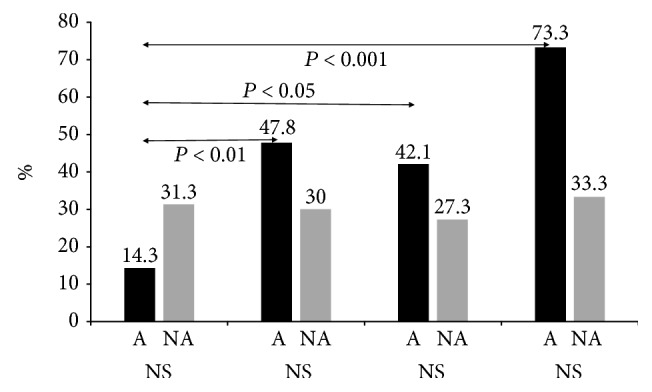
Nasal discharge eosinophils by age. A, asthma group; NA, nonasthma group. The positive nasal discharge eosinophil rates in the A group were significantly higher in patients aged 2-3 years, 4-5 years, and >6 years than in patients aged 0-1 years (*p* < 0.05 − 0.001, chi-square test).

The nasal discharge eosinophil characteristics, according to age groups, are shown in Figure 1. The positive nasal discharge eosinophil rates according to age in the A group were 14.3% (4/28) in 0-1 years, 47.8% (11/23) in 2-3, 42.1% (8/19) in 4-5 years, and 73.3% (11/15) in >6 years. The positive nasal discharge eosinophil rates increased with increasing age. The positive nasal discharge eosinophil rates in the A group were significantly higher for patients aged 2–3 years, 4–5 years, and >6 years than in patients aged 0–1 years (*p* < 0.05–0.001, chi-square test). Meanwhile, the positive nasal discharge eosinophil rates according to age in the NA group were approximately the same across all age groups (approximately 30% in each age group). A comparison of nasal eosinophil positive rates between group A and group NA revealed that these were higher in the A group across all age groups, with the exception of the 0–1-year-old age group (NS, chi-square test).

**Figure 2:**
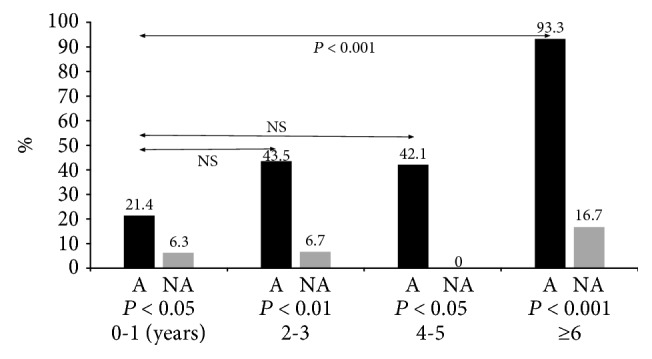
RAST-positive rates by age. A, asthma group; NA, nonasthma group. Positive RAST rates were significantly higher in the older age groups than in the 0–1-year-old age group (NS, *p* < 0.01).

The RAST-positive rates, according to age, are shown in Figure 2. The RAST reactions were positive (>0.35 UA) mainly for egg whites, *Dermatophagoides farinae*, and cat skin scraps. The positive RAST rates according to age in the A group were 21.4% (6/28) in 0-1 years, 43.5% (10/23) in 2-3 years, 42.1% (8/19) in 4-5 years, and 93.3% (14/15) in >6 years. These positive RAST rates were significantly higher in the older age groups than in the 0–1-year-old age group (NS, *p* < 0.001). The positive age-based RAST rates in the NA group were approximately 0–10% overall and did not differ significantly between each age group. According to each age group, the RAST-positive rates on the A group were significantly higher than those on the NA group (*p* < 0.05–0.001).

**Figure 3:**
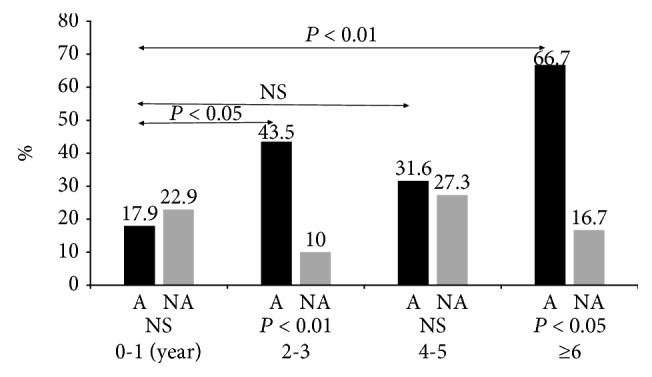
Positive allergic family history rates according to the age group. A, asthma group; NA, nonasthma group. Positive allergic family history rates were significantly higher in the older age groups than in the 0–1-year-old age group (NS, *p* < 0.01).

The positive allergic family history rates, according to age, are shown in Figure 3. The positive allergic family history rates, according to age, in the A group were 17.9% (5/28) in 0-1 years, 43.5% (10/23) in 2-3 years, 31.6% (6/19) in 4-5years, and 66.7% (10/15) in >6 years. These positive allergic family history rates were significantly higher in the older age groups than in the 0–1-year-old age group (NS, *p* < 0.01). The positive allergic family history rates in the NA group were approximately 20% overall and did not differ significantly by age group. For each age group, the positive allergic family history rates of the A group were higher than those of the age-matched NA group (NS, *p* < 0.01), except for the 0–1-year-old age group.

**Figure 4:**
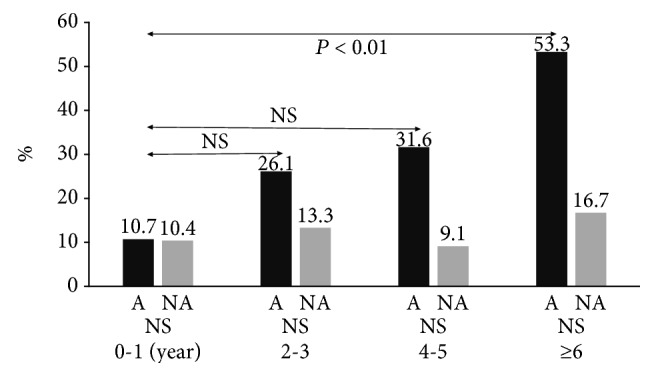
Positive coexisting or past allergic diseases rates (eczema, allergic conjunctivitis). A, asthma group; NA, nonasthma group. The rates of coexisting allergic diseases were significantly higher in the older age groups than in the 0–1-year-old age group (NS, *p* < 0.01).

The positive rates of coexisting or past allergic diseases (eczema, allergic conjunctivitis) are shown in Figure 4. The positive coexisting allergic disease (other than asthma) rates in the A group were age 0–1 years, 10.7% (3/28); age 2–3 years, 26.1% (6/23); age 4-5 years, 31.6% (6/19); and age > 6 years, 53.3% (8/15). The other coexisting allergic diseases rates were significantly higher in age > 6 years, but not significant in any other older age group, when compared to the 0-1-year-old age group (*p* < 0.01).

Each of the above 4 assessments in the A group showed significantly higher positive rates in each age group older than the 0–1-year-old age group. On the other hand, each of the above assessments in the NA group showed similar positive rates across all age groups, without significant differences. According to each age group, the positive rates of allergic family history rates on the A group were not significantly different, when compared with those of the NA group.

Correlations between nasal discharge eosinophils and other predictive factors in the A group are presented in Table 3. In the A group, no significant correlation was observed between nasal discharge eosinophils and an allergic family history. However, significant correlations were observed between nasal discharge eosinophils and the RAST results (*p* < 0.01), as well as other coexisting or past allergic diseases (*p* < 0.05). In other words, positive nasal discharge eosinophil rates were significantly correlated with RAST positivity and the presence of coexisting or past allergic diseases.

## 4. Discussion

This study evaluated nasal discharge eosinophils in infants and young children with both nasal symptoms (without fever) and recurrent respiratory symptoms. These trends were evaluated according to patient age groups to determine the utility of these factors for the prediction of persistent asthma.

### 4.1. Changes in Nasal Eosinophil Positivity Rates between Study Groups

Nasal eosinophil positive rate in the A group significantly increases with age. In the NA group, there was no significant change in the eosinophil-positive rate with an increase in age. In the other factors recognized as predictive factors for persistent asthma, similar patterns were detected.

There are two likely reasons that the positivity rates, except for an allergic family history, were significantly higher in the older age groups. Namely, as a child becomes older, negative findings at age 0–1-year-old may become positive and symptoms may be resolved. However, the ratio of patients with findings that become positive to those whose symptoms disappear, all while age increases, is unclear based on the present study. Moreover, reaching a conclusion is difficult because factors such as wheezing, cough, and dyspnea also vary with age [7].

For younger child asthma with no allergic factors, the causes of asthma symptoms are considered viral infections, GERD, among others. A previous report has shown that even if the cause is viral infection with younger childhood asthma, allergic inflammation, asthma, and chronic obstructive disease may occur [8]. A previous study by Kwiecien et al. found differences in the clinical features of childhood asthma regarding the intensity of esophageal acid exposure. Symptoms of asthma in nonatopic individuals with early onset and difficult-to-control nighttime asthma attacks suggest the possibility of concomitant, clinically relevant GERD. We excluded patients that had no treatment effect from beta2 stimulant inhalation use for nighttime asthma attacks. Therefore, in our study, patients with asthma symptoms resulting from GERD may have been excluded to some extent, but nasal eosinophil-negative patients with asthma symptoms have the possibility of becoming persistently (allergic) asthmatic.

RAST positivity, other coexisting allergic diseases, and the percentage of peripheral blood eosinophils may also change with age, whereas a positive or negative allergic family history is unlikely to be influenced by older age. Because most family histories represent predominately the parent's medical history, those with allergic histories are already of adult age. A correlation between a positive family history and older age may indicate that respiratory symptoms are delayed in a patient with a positive family history or that respiratory symptoms have disappeared in a patient with a negative family history.

In the nonasthma group, none of the studied factors had significant changes with age group, which indicates there is no capacity for each allergy to increase with patient age. This result also indicates that eosinophil-positive rhinitis morbidity without asthma symptoms does not change with age.

### 4.2. Factors for Persistent Asthma

Bronchial asthma in children is a multifactorial disease [7], and these multiple factors differ according to patient age. In infants and young children, asthma is often a nonallergic disease in which wheezing is caused by a viral infection or GERD [2], and this often resolves spontaneously before these children begin elementary school. In contrast, infants and young children who have asthma associated with allergic factors show an increased risk of persistent asthmatic symptoms, even after they enter elementary school [9].

The 2016 Global Initiative for Asthma guidelines state that other coexisting or past allergic diseases, such as eczema and an allergic family history, are prognostic features in asthma [2]. Elevated serum IgE levels and the probability of RAST positivity also increase with age [5]. In addition, among patients with severe eczema and persistent atopic dermatitis without respiratory symptoms, those with persistent infantile atopic dermatitis are more likely to develop asthma by the age of 6 [10].

A 2007 report on the National Institutes of Health website [3] also states the following: “The asthma predictive index generated identifies the following predictive factors for developing persistent asthma among children younger than 3 years of age who had four or more episodes of wheezing during the previous year: either (1) one of the following: parental history of asthma, a physician diagnosis of atopic dermatitis, or evidence of sensitization to aeroallergens, or (2) two of the following: evidence of sensitization to foods, ≥4 percent peripheral blood eosinophils, or wheezing apart from colds.”

An allergic family history, other coexisting or past allergic diseases, and RAST positivity have been reported as predictive factors in asthma [11], but the presence of nasal discharge eosinophils as a predictive factor has not been reported. Knowledge about the prognosis of childhood asthma is very important for attending physicians who treat these patients because the prognosis for persistent asthma and its severity may lead to different treatment strategies [12].

The pathogenesis of childhood allergic asthma is thought to involve airway eosinophilic inflammation [1], but detecting eosinophils in the airway can be challenging. Fiber optic bronchoscopy in older children with bronchial asthma showing bronchial mucosal hypertrophy and eosinophilic infiltration has been reported [1], but similar findings on bronchoscopy in infants aged 0–1 years have rarely been reported.

### 4.3. Relationship of Eosinophil and Predictive Factor for Persistent Asthma

There are some reports about the relationship between nasal eosinophil and childhood allergic diseases. Lozano et al. [12] described that eosinophil count in the nasal cytology was useful to differentiate rhinitis with a positive allergen skin test from rhinitis patients with negative skin tests. Another study described that not only sputum eosinophils but also FENO is important for the risk assessment of patients with asthma in future studies [13]. In our study, almost all nasal eosinophil counts were less than 1+ (stage 2 of 4), especially in younger children. There is a similar study reporting that low-grade disease activity in early life precedes childhood asthma and allergy [14]. It has been reported that early-life sensitivity is associated with allergic eosinophilic rhinitis at 4 years of age [15], which is similar to our results, but the approach in this report was different from our study.

## 5. Conclusions

Testing for nasal discharge eosinophils is easy, inexpensive, and similar to other predictive factors, with higher positive rates in older age groups. Moreover, a correlation was observed between nasal discharge eosinophils and other allergic disease markers (RAST positivity and coexisting diseases). However, the relationship between nasal eosinophil and RAST is not clear. There were patients (*n* = 11) who were positive for nasal eosinophil, but negative for RAST. It is considered that these patients are potentially RAST-positive as age increases. Therefore, testing for nasal discharge eosinophils can be useful in the diagnosis of childhood allergic asthma. Nasal discharge eosinophils should also be considered a predictive factor for persistent asthma in these children. A further study with a large number of patients may be necessary. The data of this study was shared with the data of the data journal (Data in Brief) [16].

## Figures and Tables

**Table 1:** The number of patients in each age group.

Age (y)	0-1	2-3	4-5	>6	Sum	Total
Asthma	28	23	19	15	85	180
No asthma	48	30	11	6	95	(M: 94, F: 86)

**Table 2:** Nasal eosinophil counts for each group.

Eosinophil count	0	<5/10F	5-50/10F	50-100/10F	>100/10F
By microscope (400x)		+−	+	++	+++
Asthma (%)	60.0	12.9	20.0	2.3	4.7
No asthma (%)	69.4	16.8	11.6	1.1	1.1

An eosinophil count 0/10F was considered negative, and eosinophil counts greater than 1/10F were considered positive.

**Table 3:** Match correlations between nasal eosinophils and other predictive features.

	RAST		Family		Other	Allergy
	Number of patients		Number of patients		Number of patients	
	Negative	Positive	Negative	Positive	Negative	Positive
*A group*						
Nasal eosinophil (+)	11	23^∗∗^	18	16	20	14^∗^
Nasal eosinophil (−)	36	15	36	15	42	9
*NA group*						
Nasal eosinophil (+)	27	2	23	6	24	5
Nasal eosinophil (−)	62	4	54	12	60	6

A, asthma group; NA, nonasthma group; family, atopic family history; other, other allergic diseases. ^∗^*p* < 0.05, ^∗∗^*p* < 0.01, chi-square test.

## Data Availability

The data of this study was shared with the data of the data journal (Data in Brief) [16].
